# Push and Pull: What Factors Attracted Applicants to Emergency Medicine and What Factors Pushed Them Away Following the 2023 Match

**DOI:** 10.5811/westjem.21249

**Published:** 2025-02-14

**Authors:** Michael Kiemeney, James Morris, Lauren Lamparter, Moshe Weizberg, Andy Little, Brian Milman

**Affiliations:** * Loma Linda University School of Medicine, Loma Linda, California; † Texas Tech University Health Sciences Center, Lubbock, Texas; ‡ University of Illinois Chicago, Chicago, Illinois; § Staten Island University, Staten Island, New York; ∥ AdventHealth East Orlando, Orlando, Florida; ¶ University of Texas Southwestern Medical Center, Dallas, Texas

## Abstract

**Introduction:**

Emergency medicine (EM) historically enjoyed a nearly 100% match rate. A rapid change saw 46% of EM programs with one or more unfilled positions after the 2023 Match. Much has been discussed about potential causes, and characteristics of unfilled programs have been investigated. We surveyed recent applicants to EM to further understand what continues to draw them to EM and what concerns deter them from choosing a career in EM.

**Methods:**

A cross-sectional, mixed methods survey was distributed in the summer of 2023 to a convenience sample of respondents via the listservs of national EM resident and student organizations as well as clerkship directors in EM. We did not calculate response rate due to listserv convenience sampling. A total of 213 responses were received, representing 7.7% of the total number of EM applicants (2,765) in 2023. Applicants were asked to rank from 1 to 5 their experiences with EM and the characteristics of the specialty that were important in their career decision. We calculated means and 95% confidence intervals for quantitative results. We performed qualitative analysis of free-text responses to identify themes.

**Results:**

Positive factors for applicants were interactions with EM faculty (4.29 on 1–5 scale) and residents (4.42) as well as clinical experiences in third-year (4.53) and fourth-year clerkships (4.62). Applicants continue to be drawn to EM by the variety of pathology encountered (4.66), flexible lifestyle (4.63), and high-acuity patient care (4.43). Most applicants (68.5%) experienced advisement away from EM. Of those who received negative advisement, non-emergency physicians were the most common source (73.3%). Factors negatively influencing a career choice in EM were corporate influence (2.51), ED crowding (2.52), burnout (2.59), presence of advanced practice practitioners (APP) in EM (2.63), and workforce concerns (2.85). Job concerns stemming from the 2021 EM workforce report were identified by respondents as the primary reason for recent Match results.

**Conclusion:**

Applicants noted clinical experiences in the emergency department and interactions with EM attendings and residents as positive experiences. High-acuity patient care, variety of pathology, and flexible lifestyle continue to attract applicants. Applicants identified EM workforce concerns as the primary contributor to recent EM Match results. Corporate influence, ED crowding, burnout, and presence of APPs in the ED were also significant issues.

Population Health Research Capsule What do we already know about this issue?

*Applicant and specialty characteristics attracting applicants to EM have been previously documented.*

What was the research question?

*What factors deterred and attracted applicants to EM during the 2023 Match?*

What was the major finding of the study?

*The 4th-year clerkship was the major attracting factor (mean 4.62, 95% CI 4.50–4.74), while corporate influence (mean 2.51, 95% CI 2.33–2.69) was the strongest deterring factor.*

How does this improve population health?

*These findings offer new insights into applicant perspectives of EM and specialty-choice considerations following the 2023 Match.*

## INTRODUCTION

Emergency medicine (EM) has historically enjoyed a very competitive outcome in the National Residency Matching Program (NRMP, or “the Match”) with >95% of programs filling their spots.^1^ Beginning in 2022, however, a dramatic decline occurred leaving many programs unfilled.^2^ This decline continued in 2023, with 46% of EM programs remaining unfilled.^3^ Although 79.1% of those programs filled in the Supplemental Offer and Acceptance Program (SOAP),^4^ this represents a tremendous change from previous years.

The cause of this change is likely multifactorial, with major contributing factors being the expansion of the number of residency positions, student perceptions of the future job market within EM, and the virtual interview format.^5^ ^,^ ^6^ Other proposed etiologies of the decline include the corporate practice of EM (which occurs when a non-physician or corporation exerts control over the medical decision-making or collects reimbursement for the medical services of physicians),^7^ the expanded use of advanced practice practitioners (APP) such as physician assistants and nurse practitioners in the emergency department (ED), and increased burnout following a global pandemic.^6^ Concerns regarding the job market and expanded use of APPs are likely related to the 2021 EM workforce report by Marco et al, which proposed a range of potential outlooks based on various factors with the most publicized result being a projected oversupply of emergency physicians by 2030.^8^

Several factors affected which programs were more likely to go unfilled in the Match. Gettel et al found that programs accredited within the previous five years, as well as programs that were under for-profit ownership were more likely to go unfilled.^9^ Another study found that predictors of not filling were having unfilled positions in the previous Match, a smaller program size, location in the Mid-Atlantic or East North Central area, prior American Osteopathic Association accreditation, and corporate ownership structure.^10^ Overall, programs felt their match outcomes were worse than in previous years, but they perceived the quality of applicants as similar to previous years.^5^

Many factors influence a student’s decision on which specialty to pursue including role models, financial incentives, gender, degree of patient contact, procedural skills, prestige, and lifestyle.^11^ ^–^ ^14^ The factors most associated with a choice to specialize in EM include lifestyle, diversity of patient presentations, flexibility in choosing a practice location, work-life balance, and perceived job satisfaction.^15^ ^–^ ^19^ Factors associated with earlier selection of EM include early exposure to the field, presence of an EM residency program at a student’s medical school, prior employment in the ED, previous experience as a prehospital practitioner, and completion of a third-year EM clerkship.^16^

In this study we surveyed EM applicants from 2022 and 2023 to identify factors deterring or attracting them to the specialty as well as modifiable influences impacting their career decisions. To restore the competitive nature of EM in the Match, it is important to know what motivates medical students to select EM as a specialty in the current environment. It is additionally important to further understand the factors contributing to decreased interest in EM, so that we can continue to address these as a specialty.

## METHODS

The project was conceived by the Council of Residency Directors in Emergency Medicine (CORD) Match Task Force, which includes representatives from the American Academy of Emergency Medicine (AAEM), American Academy of Emergency Medicine Resident and Student Association (AAEM/RSA), American College of Emergency Physicians (ACEP), American College of Osteopathic Emergency Physicians (ACOEP), ACOEP Resident and Student Organization (ACOEP RSO), Association of Academic Chairs in Emergency Medicine (AACEM), CORD, Emergency Medicine Residents’ Association (EMRA), the Society for Academic Emergency Medicine (SAEM), and SAEM Residents and Medical Students (SAEM RAMS). Task force members collaborated to design the survey instrument. The conclusions in this paper represent the views and opinions of the individual authors and do not represent the views of the organizations. The study was approved by the Loma Linda University Health Institutional Review Board.

We performed a literature review using PubMed to collect studies investigating factors impacting residency applicants’ specialty choice. Questions were adapted from prior published studies.^16^ ^,^ ^20^ Current factors not previously investigated, such as COVID-19 or EM workforce projections, were added following an iterative process of consensus development within the research group. The survey was reviewed by the CORD Match Task Force members and edited. The survey was then pilot-tested by current medical students and residents. We analyzed the responses, and the survey was revised for clarity and brevity following the beta respondents’ feedback.

Medical students were asked multiple-choice questions regarding their residency application strategy including whether they had applied to more than one specialty and, if so, which specialties they applied to. The survey participants were asked to rank specialty characteristics influencing their choice of EM as a career on a five-point Likert scale from strongly positive to strongly negative. They were also asked to rank the impact of prior experiences on their specialty choice on a five-point Likert scale from very positive to very negative. We investigated the impact of career advisement using multiple-choice questions with the option to select up to three responses. Finally, free-text response questions were asked to assess applicants’ opinions about the causative factors leading to the 2023 EM Match results. Comment in this space was optional and not meant to reach saturation of themes; rather, it was meant to provide participants the opportunity to give additional details about their experiences.

We used a convenience sample of EM-bound medical students who applied in both the 2022 and 2023 Match and those who considered or are considering applying to EM in upcoming Match cycles. Survey respondents were sent a web-based survey via Qualtrics (Qualtrics International, Inc, Seattle, WA) in the summer of 2023. Reminder messages were distributed monthly during the data collection period. The survey was distributed through the listservs of current medical students interested in EM as identified by their membership in an EM national organization including AAEM/RSA, ACOEP RSO, EMRA, and SAEM RAMS. Surveys were also distributed through the SAEM Clerkship Directors in Emergency Medicine (CDEM) listserv to be sent to their recently matched applicants who matched into EM or had considered but ultimately decided not to pursue EM. Convenience sampling via listserv distribution did not allow for survey distribution quantification or response-rate calculation. Comparing the number of survey responses (213) to the number of applicants to EM in the 2023 Match (2,765) shows our survey responses were equal to 7.7% of the total number of EM applicants in 2023. The intended survey participants included medical students who 1) considered but ultimately did not apply to EM residency; 2) applied to EM as their only specialty choice; 3) dual applied to EM and an alternate specialty choice; or 4) entered EM through the SOAP.

A financial incentive of a $10 electronic gift card was given to the first 160 participants. Financial support for the study was provided by AAEM, AAEM/RSA, ACEP, ACOEP, AACEM, CORD, and SAEM.

We analyzed data using Microsoft Excel 365 (Microsoft Corporation, Redmond, WA) to calculate means and percentages. We calculated 95% confidence intervals (CI) using an online tool.^21^ A phenomenological approach to qualitative analysis was used and free-text responses were coded by two authors with experience in qualitative analysis (JM, BM) after establishing a codebook through an iterative process to generate an understanding of the phenomenon of the EM match process in concert with the quantitative questions. Any disagreements between codes were resolved by a third author (MK).

## RESULTS

We received responses from 213 individuals. Demographics are shown in Table 1. Most respondents (92.8%) had applied to residency already. Of those, 87.2% applied to EM in the Match. Respondents secured an EM residency position in the 2023 Match (69.5%), 2022 Match (9.6%), 2023 SOAP (12.3%), 2022 SOAP (0.5%), and by other means (5.3%). A small proportion of respondents (2.7%) were not entering EM residency.

**Table 1.** Demographic data of survey respondents.

Characteristics	
Age (years) (n = 173)	N (%)
<25	1 (0.6%)
25–29	108 (62.4%)
30–34	47 (27.2%)
35–39	13 (7.5%)
40–44	2 (1.2%)
>44	2 (1.2%)
Gender identity (n = 173)	
Male	99 (57.2%)
Female	69 (39.9%)
Non-binary/third gender	1 (0.6%)
Prefer not to say	4 (2.3%)
Race (n = 177)	
American Indian/Alaska Native	1 (0.6%)
Asian	20 (11.3%)
Black/African American	10 (5.6%)
Hawaiian/Pacific Islander	0
White	132 (74.6%)
Other	8 (4.5%)
Prefer not to say	6 (3.4%)
Ethnicity (n = 173)	
Hispanic/Latino	18 (10.4%)
Not Hispanic/Latino	147 (85.0%)
Prefer not to say	8 (4.6%)
Medical school background (n = 211)	
MD in US	85 (40.3%)
DO in US	90 (42.7%)
US citizen IMG	28 (13.3%)
Non-US citizen IMG	8 (3.8%)
Medical school type (n = 171)	
Private	103 (60.2%)
Public	67 (39.2%)
Other	1 (0.6%)
Medical school geographic region (n = 171)	
Central (IA, IL, IN, KS, MI, MN, MO, MT, ND, NE, OH, SD, WI)	43 (25.1%)
Northeast (CT, DC, DE, MA, MD, ME, NH, NJ, PA, RI, VT)	29 (17.0%)
South (AL, AR, FL, GA, KY, LA, OK, MS, NC, SC, TN, TX, VA, WV)	70 (40.9%)
West (AK, AZ, CA, CO, HI, ID, NM, NV, OR, UT, WA, WY)	29 (17.0%)

*IMG*, international medical graduate; *MD*, Doctor of Medicine; *DO*, Doctor or Osteopathic Medicine.

In comparison to applicants securing a position in the 2023 Match, our sample was fairly similar with regard to gender breakdown (57.2% male, 39.9% female in our sample vs 54.8% male, 45.2% female in the Match) but oversampled osteopathic seniors (42.7% in our study vs 24.3% in the Match). Regarding application strategy, 70.1% applied to only EM residencies. Some individuals applied to more than one specialty with EM preferred (12.3%). The most common secondary specialties were internal medicine and family medicine. Applying to EM as the secondary specialty occurred in 2.1% of individuals with primary specialties being anesthesiology, interventional radiology, orthopedic surgery, and physical medicine and rehabilitation. Respondents who chose not to apply to EM at all made up 13.4% of responses. This group of individuals most commonly chose to apply to anesthesiology (39.1%), orthopedic surgery (17.4%), general surgery (17.4%), family medicine (13.0%), internal medicine, pathology, and preliminary year (each 8.7%). (Response option was “Select all that apply,” response sum >100%).

Applicants most commonly chose to apply to EM in the third year of medical school (33.5%) or before medical school (33.0%). The remaining responses were evenly split among the pre-clinical years of medical school (6.8%), the fourth year of medical school (8.9%), after medical school (6.8%), and during SOAP (8.4%). Participants were exposed to EM in their medical school via required EM clerkships in the fourth year (42.1%), required clerkships in the third year (24.0%), EM electives in the fourth year (17.0%), and EM electives in the third year (11.1%). Table 2 shows the degree of influence each factor held in the applicants’ choice of EM as a career. The most frequently cited positive influences were EM residents on shift (4.42 on a 1–5 scale), EM attendings on shift (4.29), the fourth-year EM clerkship (4.62), and third-year EM clerkship/elective (4.53). Prior experience in the ED in a non-physician role (4.43), in emergency medical services (EMS) (4.52) or as a scribe (4.55), were identified less frequently but as very positive factors.

**Table 2.** Factors influencing selection of career in emergency medicine.

What factors influenced your choice of EM as a career?	Strongly positive (5)	Somewhat positive (4)	Neutral (3)	Somewhat negative (2)	Strongly negative (1)	Mean (95% CI)
4^th^-year EM clerkship	118	26	8	3	2	4.62 (4.50, 4.74)
Worked as scribe in ED	40	12	5	0	1	4.55 (4.35, 4.75)
3^rd^-year EM clerkship/elective	79	27	5	2	3	4.53 (4.37, 4.69)
Worked in EMS outside hospital	32	7	2	2	1	4.52 (4.24, 4.80)
Shadowing experience in ED	44	24	5	3	0	4.43 (4.25, 4.61)
Worked non-physician role in ED	24	10	5	1	0	4.43 (4.18, 4.68)
ED residents on shift	81	52	14	1	1	4.42 (4.30, 4.54)
Other	8	3	0	0	1	4.42 (3.79, 5.05)
Family/friend is emergency physician	33	27	10	1	0	4.30 (4.12, 4.48)
ED attending on shift	75	61	13	4	3	4.29 (4.15, 4.43)
Mentor/advisor	54	35	12	6	2	4.22 (4.04, 4.40)
Volunteer experience in ED	22	21	9	1	0	4.21 (4.00, 4.42)
EM experience in preclinical years	37	28	16	5	0	4.12 (3.92, 4.32)
EM related research	17	21	24	1	1	3.81 (3.59, 4.03)
Word of mouth/reputation	22	44	22	14	8	3.53 (3.31, 3.75)

*CI*, confidence interval; *EM*, emergency medicine; *ED*, emergency department; *EMS*, emergency medical services.

Job concerns/workforce report (65.8%), burnout (56.7%), increased use of advanced practice practitioners (APP) (50.8%), and corporate influence in EM (42.5%) were the most-cited reasons for advising applicants away from EM. Emergency department crowding (12.5%) and EM experience during the COVID-19 pandemic (5.8%) were less commonly cited concerns. Participants were asked about advisement and its influence on their specialty choice: 68.5% reported being advised against choosing EM residency training. The most common sources of advisement away from EM were attendings/residents in non-EM specialties (73.3%), peers (50.0%), social media/message boards (47.5%), and EM attendings (37.5%). Medical school representatives in the Dean’s office accounted for a small proportion of advisement away from EM (15.8%). Most participants in our survey (81.8%) reported that advising against entering EM did not change their application strategy. Of those who initially pursued a different specialty 5.7% ultimately entered EM in the SOAP, 5.0% applied to another specialty as a backup to EM, and 3.3% applied to EM as a backup specialty. Of those applicants who did not change application strategy despite negative advice about EM, the most commonly cited reasons were perceived fit with EM (73.7%), flexible lifestyle of EM (64.6%), lack of interest in other specialties (49.5%), and doubt in accuracy of workforce report (49.5%).

Very few participants said they would not advise a friend to apply to EM for the 2024 Match (2.3%). Most (75%) would advise a friend to choose EM. Most of those who indicated they would advise a friend against applying to EM would do so because of concerns about fit for the specialty (42.9%) and the job market (22.9%), with corporatization of medicine, APP expansion, and burnout also mentioned.

Most somewhat agreed or strongly agreed that their peers would be more interested in EM as a career if they were exposed to EM during a rotation in the third year or earlier (82.7%). Participants were asked what they thought would make EM more appealing to peers who were undecided about a specialty but were considering EM. The most common responses included early exposure to EM (31.5%) and alleviating concerns about job security raised by the EM workforce report (30.2%). Other suggestions included addressing the expanded use of APPs in the ED (10.1%), improving the perception of EM among medical students and physicians (9.4%), and improving work-life balance and compensation (8.7% and 8.1%, respectively).

Table 3 shows how applicants ranked different factors when choosing EM as a career. The most important positive factors were variety of patient pathology (4.66 on a 1–5 scale), lifestyle/flexibility (4.63), high-acuity patient care (4.43), length of residency training (4.37), and family considerations (4.36). Participants were asked specifically if they believed that EM is a “lifestyle specialty,” and 60.1% responded yes; 9.0% did not consider EM a lifestyle specialty, while 28.1% were neutral, and 2.8% were unsure. The factors negatively influencing a career choice in EM, defined as 95% CI less than 3.0, were corporate influence in EM (2.51, 2.33–2.69), ED crowding (2.52, 2.37–2.67), burnout (2.59, 2.44–2.74), and use of APPs in EM (2.63, 2.47–2.79). Average rating of concerns about EM experience during the COVID-19 pandemic (2.95) and workforce report/job security was negative (2.85); however, upper limit of 95% CI was positive, 3.12 and 3.03, respectively.

**Table 3.** Importance of various aspects of emergency medicine to applicants in the 2023 Match.

How important were the following factors in your decision to apply to EM residency	Strongly positive (5)	Moderately positive (4)	Neutral (3)	Moderately negative (2)	Strongly negative (1)	Does not apply	Mean (95% CI)
Variety of pathology	132	24	16	1	0	5	4.66 (4.56, 4.76)
Lifestyle/flexibility	124	39	11	1	0	3	4.63 (4.54, 4.72)
High-acuity patient care	101	47	24	1	0	5	4.43 (4.32, 4.54)
Length of residency training	89	62	21	2	0	4	4.37 (4.26, 4.48)
Family considerations	95	52	22	5	0	4	4.36 (4.24, 4.48)
Compensation/salary	57	79	27	10	0	5	4.06 (3.93, 4.19)
Mentor/advisor influence	61	55	40	7	2	13	4.01 (3.87, 4.15)
Fellowship options	44	56	59	5	4	10	3.78 (3.64, 3.92)
Availability of jobs in desired location	41	67	40	19	4	7	3.71 (3.56, 3.86)
Competitiveness of EM match	30	47	83	6	3	9	3.56 (3.43, 3.69)
Student debt	18	54	70	11	3	22	3.47 (3.34, 3.61)
Career longevity	29	42	59	38	5	5	3.30 (3.14, 3.46)
COVID-19 experience in EM	20	24	69	39	17	9	2.95 (2.78, 3.12)
EM workforce report/job security	20	21	59	48	19	11	2.85 (2.68, 3.03)
APPs in EM	11	17	64	47	27	12	2.63 (2.47, 2.79)
Burnout in EM	13	12	57	75	17	4	2.59 (2.44, 2.74)
ED crowding	8	12	67	56	27	8	2.52 (2.37, 2.67)
Corporate influence in EM	16	14	48	50	39	11	2.51 (2.33, 2.69)

*APPs*, advanced practice practitioners; *CI*, confidence interval; *EM*, emergency medicine; *ED*, emergency department.

Applicants were asked to identify the most important reason contributing to a larger-than-normal number of unfilled positions in the EM Match. They identified concerns about job security and the future EM workforce as the primary concern (Table 4). Qualitative responses to the increase in unfilled spots in the EM Match predominantly reflected concerns regarding the EM workforce report and job security. Themes and representative quotations are included in Table 5.

**Table 4.** Single most important reason for unfilled emergency medicine (EM) residency positions in 2022 and 2023 Match, per EM applicants.

Response	N%
Workforce/job security	79 (53.0%)
COVID-19	28 (18.8%)
Number of residencies	20 (13.4%)
Burnout	17 (11.4%)
APP expansion	15 (10.1%)
Perception of emergency medicine	15 (10.1%)
Quality of life, change in practice environment (boarding, volumes, etc)	11 (7.4%)
Corporatization	8 (5.4%)
Other	6 (4.0%)
Programs’ failure to adapt to changing applicant pool	2 (1.3%)

Note: Totals exceed 100%, as respondents could indicate more than one item; % indicates the percent of total respondents endorsing a choice.

*APP*, advanced practice practitioner; *EM*, emergency medicine.

**Table 5.** Qualitative analysis themes and representative quotations regarding the 2022 and 2023 EM match.

Theme	Code	Guideline for use
**Employment opportunities**	**Workforce/job security**	**This code is used when participants discuss the workforce report, job security, employment opportunities, or difficulty finding jobs**
• *There is a myth going around that there are not enough jobs for EM physicians after residency. I know a lot of people that made this comment upon saying I was applying to EM* • *Covid, and that damn memo. Yall shot yourselves in the damn foot with that bonehead move* • *Workforce report hysteria* • *The infamous report predicting a coming labor surplus. The timing lines up and it tracks with what friends in med school were saying*		
	**Number of residencies**	**This code is used when participants discuss residency expansion**
• *Increased amount of residency program spots created by CMG hospitals* • *Too many residency programs* • *Surplus of “pop-up” programs leveraging resident labor with no intention of real training*		
	**APP expansion**	**This code is used when participants discuss competition with APPs for employment or increased use of APPs in EM**
• *Midlevel creep* • *increasing number of NPs/PAs filling in positions* • *PA/NP takeover* • *Increased NP/ PA replacing jobs and then MD license online for anything they do. Including signing their charts*		
**Practice environment**	**Burnout**	**This code is used when participants discuss burnout**
• *Concern over burnout* • *Fear of burnout* • *Emergency doctors burnt out*		
	**COVID-19**	**This code is used when participants discuss the impact of COVID-19**
• *Treatment during COVID-19* • *COVID-19 experiences, lack of patient care opportunities during COVID-19* • *High stress, especially during COVID-19* • *COVID-19 showed EM’s true colors* • *COVID-19 experiences and fears of future health risks*		
	**Corporatization**	**This code is used when participants discuss corporatization of emergency medicine or private equity influence**
• *Corporate takeover, thus physicians lose power every day* • *Corporate practice of medicine* • *HCA programs!!!!! There are a ton of new, sketchy programs.* • *Increase in for-profit hospital slots available in Texas, Cali, and Florida*		
	**Quality of life, change in practice environment (boarding, volume, etc.)**	**This code is used when participants discuss negative practice factors**
• *Lack of perceived quality of life* • *Bad job prospects and ED culture has become toxic* • *Seeing patients in waiting rooms/bed holds* • *Culture of what EM has become. No one wants to choose to work in this over run environment especially when the job market is uncertain when there are specialties like dermatology and sub- specialties where you don’t have to deal with the chaos and patient volumes we are now seeing in the ED. ER medicine is at an all-time low and never used to be this overwhelming pre-pandemic.*		
**Applicant or match factors**	**Programs’ failure to adapt to changing applicant pool**	**This code is used when participants discuss residency programs’ failure to assess competitiveness or select applicants efficiently**
• *Mismatch between programs’ opinion of themselves/how they are perceived vs actual applicant perceptions of programs.* • *Programs being overly selective and not honestly introspecting regarding how applicants perceive their program*		
	**Perception of emergency medicine**	**This code is used when participants discuss negative perceptions of emergency medicine among students or through social media or mentors**
• *Lack of respect to emergency physicians and thought that we are not that smart* • *Perception from attendings of both EM and non-EM* • *Social media influence and immaturity on behalf of applicants* • *Decreased perceived competitiveness leading to lack of interest* • *Bad reputation among consultant specialties* • *Jack of all trades/EM incompetency stigma*		

*APP*, advanced practice practitioner; *CMG*, contract management group; *EM*, emergency medicine; *ED*, emergency department; *HCA*, Hospital Corporation of America; *NP*, nurse practitioner; *PA*, physician assistant.

## DISCUSSION

Applicants in our survey were drawn to EM by clinical experiences in the ED during the third and fourth year and by interactions with ED residents and attending physicians during those experiences. Unfortunately, only a small proportion of applicants in our survey had required EM clinical experience during the third year of training. Developing best practice recommendations for early exposure to EM during medical school may be an area to target to increase interest in future applicants. Additionally, employment in an EM-related field (ie, EMS, scribe) prior to medical school was also a positive experience. Early identification of those students with prior EM-related employment may be an area for mentorship efforts by EM advisors.

Applicants continue to be drawn to the high-acuity patient care, diverse patient pathology, and the flexible lifestyle EM offers. These findings are in line with prior studies of EM applicant attitudes and the cornerstone of EM’s appeal.^12^ ^–^ ^19^ ^,^ ^23^ Additional factors that appeal to applicants are the variety of fellowship options available after EM residency, the length of residency training, compensation, and availability of jobs in their desired location. Family considerations are important to applicants and, coupled with the desire for a flexible lifestyle, signal a desire for work-life balance. Shift work in the ED has downsides such as sleep transitions associated with night shifts and working weekends and holidays. However, applicants were signaling those issues are still favorable to being on call or working in a clinic five days a week. Highlighting the factors that resonate with applicants is a good starting point when promoting the specialty.

With regard to factors pushing applicants away from EM, most applicants experienced badmouthing of EM and advising away from the specialty. In prior studies, over three-quarters of respondents reported experience with badmouthing of another specialty and one-quarter changed their specialty choice because of it.^24^ ^–^ ^26^ When uncertain applicants are narrowing their specialty choices between a few serious options, contending with negativity about your career choice, both now and in the future, from friends or mentors in other specialties may be enough to sway someone away from EM.

The most common source of advice against EM in 2023 was not from peers, formal mentors, or Dean’s offices but from attendings and residents in non-EM specialties. Experiencing negative advisement from a trusted mentor about one’s desired specialty is likely impactful. In addition, applicants reported receiving negative pressure from their peers and social media. Most people involved in EM medical education suspected applicants were being advised away from EM. This was suggested by our data. Most assumed advisors from the Dean’s office were advising students away from EM toward more prestigious specialties or those with safer match rates. But that was not the case in our survey, as advisors in the Dean’s office ranked as the sixth most frequent source of advisement away from EM.

Additional factors pushing applicants away from EM were corporate influence in EM, ED crowding, burnout, the use of APPs in EM, the experience of emergency physicians during COVID-19, and concerns regarding job security stemming from the 2021 EM workforce report. Applicants are wary of entering a specialty dominated by corporations that place profits over patient care. Residencies at for-profit clinical sites had 1.3 times greater risk of not filling in 2023.^9^ Applicants are showing an aversion to training at these sites. However, spots continue to fill during the time-limited SOAP as unmatched applicants are likely excited about the ability to secure any training position. Further understanding applicant concerns and the experiences of residents in for-profit programs is important and requires additional study. Likewise, understanding the experience of EM residents who enter training via the SOAP is valuable for future investigation.

Emergency department crowding not only negatively impacts quality of patient care; it also deters future emergency physicians from entering the field. Students on ED rotations see the challenges of finding space to re-evaluate patients, delays in workup, and prolonged care of patients boarding in the ED who are awaiting inpatient beds. Efforts to address boarding as well as the implementation of surge capacity plans may result in improving this factor as students consider specialty choice.

Furthermore, burnout generated the largest number of moderate or strongly negative responses. Emergency medicine is widely cited as the specialty with the highest rates of burnout.^27^ ^,^ ^28^ Requirements to promote well-being and counter burnout exist in both undergraduate (Liaison Committee on Medical Education standard 12.3)^29^ and graduate medical education (Accreditation Council for Graduate Medical Education Common Program Requirements for residency VI.C).^30^ Prior qualitative research suggests faculty modeling may influence residents’ career perspectives, indicating targeting faculty for education on well-being and burnout may yield substantial benefits for both current and prospective residents.^31^

Applicants, additionally, have concerns about the use of APPs in the ED. Many free-text responses cited “scope creep” of APPs as well as the negative impact on physician job availability as negative factors. Applicants signaled that they are paying attention to the topic of APP usage in the ED and it is an important issue to them. National leaders in EM are actively working to protect the scope of all practitioners in the ED and continue to emphasize the importance of physician-led patient care teams. Further dissemination of these advocacy efforts and the effects on our specialty would be beneficial for applicants.

Lastly, the workforce report has been frequently hypothesized as a major contributing factor to the rapid decline in EM residency applications over the last two years.^8^ Applicants to EM in our survey confirmed this hypothesis, citing projections stemming from the report as the most important factor leading to the significant rise in unfilled EM residency positions in the 2022 and 2023 Matches. Subsequent studies have addressed workforce considerations such as physician attrition and geographic distribution.^32^ ^,^ ^33^ Further investigation and clarity into the future EM workforce would aid applicants as they weigh their career decisions.

Reinforcing the positive aspects of EM while addressing the negative factors above will go a long way toward bolstering the EM applicant pool and future workforce. The 2023 EM Match was unprecedented with 554 unmatched positions. However, EM still matched 2,456 applicants, the fourth largest number in the 2023 Match.^3^ Our survey yields insights into the positive aspects of EM that draw applicants to the specialty and identifies negative factors following the 2023 EM Match.

## LIMITATIONS

Our survey may be impacted by selection bias as our distribution method did not guarantee that every residency applicant who considered applying to EM residency was included. For this reason, survey response rate was not calculated, and it is unknown to what extent our results are representative of all EM residency applicants in the 2022 and 2023 Match cycles. Additionally, recall bias may also contribute as responses from applicants who matched to EM in 2022 were included. As potential survey participants were identified through their membership in national EM resident and student organizations, this study may not be representative of individuals who considered EM early in their medical school career and ultimately did not pursue EM. The exact number of individuals who received the survey solicitation is not known, making it impossible to calculate a response rate. Our survey responses represent 7.7% of the total number of applicants to EM in 2023, although it is unlikely the survey reached all applicants in the pool. Future studies may benefit from a longitudinal approach soliciting EM interest-group participants in the first two years of medical school and following them through their respective Match years to improve response rate.

## CONCLUSION

The specialty of emergency medicine experienced a sharp increase in unfilled positions in the 2022 and 2023 matches. Most applicants received advisement away from EM with the most common source being physicians in non-EM specialties. Applicants perceive corporate influence in EM, ED crowding, burnout, influence of advanced practice practitioners in EM, and workforce concerns as driving forces behind the EM Match results. Applicants cited clinical experiences in the ED and interactions with EM attendings and residents as positive factors. High-acuity patient care, diverse patient pathology, and flexible lifestyle were seen as positive characteristics of a career in EM.
